# Health-economic burden of dementia in South Korea

**DOI:** 10.1186/s12877-021-02526-x

**Published:** 2021-10-13

**Authors:** Changwoo Shon, Hyejung Yoon

**Affiliations:** Department of Urban Society Research, The Seoul Institute, 57 Nambusunhwan-ro, 340-gil, Seocho-gu, Seoul, 06756 Korea

**Keywords:** Dementia, Alzheimer’s disease, Burden of disease, Health-economic cost, Cost of illness

## Abstract

**Background:**

This population-based study estimated the health-economic costs of dementia from a societal perspective using nationally representative data from 2015 to 2019 and analysed recent trends in Korea.

**Methods:**

The prevalence of and mortality due to dementia were calculated using the National Health Insurance claims data and population census. The health-economic burden due to dementia was estimated using a prevalence-based approach, including the number of dementia patients and the number of deaths resulting from dementia during 2015–2019. The health-economic burden was presented separately as the national burden and the burden per capita by summing the direct and indirect costs.

**Results:**

Between 2015 and 2019, the prevalence of dementia among the elderly aged 65 years or older based on clinical diagnosis increased from 5.9 to 7.3%, with approximately 588000 elderly dementia patients in Korea. The total health-economic cost of dementia increased by about 1.5 times in the last 5 years and was estimated to be about USD 4218 million. Direct costs were 52.0% in 2019, and the proportion has been steadily increasing over the past 5 years; indirect costs accounted for 48.0% of the total burden, mainly from family members and caregivers. The health-economic cost per capita due to dementia was approximately USD 6957.

**Conclusions:**

The burden of dementia in Korea is expected to considerably increase alongside the elderly population in the future. Health policies addressing the prevention and management of dementia should be prioritised.

## Background

Dementia is a clinical syndrome characterised by severe loss of cognitive and emotional abilities, negatively affecting daily functioning and quality of life [1]. Generally, people with dementia experience deterioration in memory, thinking, behaviour, and the ability to perform daily activities. Approximately 50 million people worldwide have dementia, with nearly 10 million new cases each year. Alzheimer’s disease is its most common form, accounting for 60–70% of dementia cases. Dementia has a physical, psychological, social, and economic impact on not only people with dementia but also their families and caregivers [2]. Generally, patients need assistance when visiting the hospital with expensive supervision and intensive care when necessary [3]. Globally, dementia-related healthcare expenses were estimated to be USD 1 trillion in 2010 [3, 4]. Additionally, the cost of care is projected to increase by approximately 85% by 2030, making it the biggest economic burden for a single disease [5].

Korea is one of the fastest ageing countries worldwide, and the increasing prevalence of dementia will put a heavy burden on Korean society. For example, the old-age dependency ratio is projected to increase from 20% to approximately 70% by 2050 [6]. Korea is predicted to become a super-aged society by 2025, only 8 years after becoming an ageing society, and the proportion of the elderly population is expected to reach 37% in 2045 [7]. The faster-ageing rate indicates that the number of patients with dementia will increase rapidly in the future. The prevalence of dementia in Korea in 2020 is estimated to be 10.25% among those over 65 years, about 830000 people. By 2050, it will increase to about 15.91%, and the population of individuals with dementia is expected to increase to approximately 3.02 million people [8].

This study aims to measure and present the socioeconomic burden caused by a specific disease in monetary units. It is used as a rationale in the process of healthcare policy decisions and setting priorities. In this respect, it is important to estimate dementia-related health and economic costs. However, estimating its socioeconomic costs is difficult due to the difference in the estimation of care settings and the health system in terms of dementia care and management, leading to differences in cost components. Accordingly, the scope of the research design was limited to the burden of dementia related to healthcare service utilisation (i.e., hospitalisation, outpatient visits, medication).

South Korea has continued to expand social support for the burden of care for dementia patients and their families since the introduction of long-term care insurance in 2008. In 2014, a special level for dementia patients (Level 5) within the long-term care insurance (LTCI) system was newly established, and in 2018, a cognition assistance level (Level 6) was introduced based on the National Responsibility Plan for Dementia [9]. Although support for dementia patients and their families is expanding mainly by LTCI, only about 40% of dementia patients receive LTCI services, which is still insufficient [10]. Therefore, in this study, the cost of medical use due to dementia was estimated. We estimated the health-economic costs of dementia using nationally representative data from 2015 to 2019 and analysed recent trends in Korea. Since this study only calculated the direct and indirect costs of dementia related to medical use, it should be noted in advance that the results may be underestimated compared with the results of previous study on social costs of dementia, including both informal and long-term care costs. In addition, since this study did not include long-term care and informal care costs, so the term health-economic costs rather than socio-economic costs due to dementia would be more appropriate.

## Methods

### Study design

Costs related to dementia were estimated using a prevalence-based approach, including the number of dementia patients and resulting deaths during 2015–2019. Due to the mandatory health insurance managed by the National Health Insurance Service (NHIS) for Korean residents, the claims data from the NHIS represent the entire population of Korea [11]. Therefore, access to NHIS claims data allowed us to estimate accurate medical expenditure for Korea as a whole.

We used the following method to estimate the health-economic burden of disease:

$$The\ health- economic\ burden= Direct\ costs+ Indirect\ costs$$

We estimated the health-economic cost of dementia from a social perspective according to Davidson and Beeri [12], which defined the components of the cost of Alzheimer’ disease. The health-economic costs of dementia are divided into direct and indirect costs. Direct costs include the medical and transportation expenses paid for dementia treatment. Additionally, the cost was estimated using a top-down approach using the NHI claims data. Indirect costs include productivity loss of dementia patients and their family caregivers related to medical care utilisation. We employed the human capital approach to quantify the productivity loss of the patients and their informal caregivers. Indirect costs do not incur an actual cost but include indirect productivity losses caused by dementia. In this study, according to the human capital approach, calculated indirect costs include productivity loss due to dementia treatment and premature death and opportunity costs paid by informal care providers such as families for caring for dementia patients.

All costs were calculated by sex and age group (5 years old), and the results were divided into three age groups: under 64 years, 65 to 74 years, and over 75 years. Meanwhile, for data related to medical use of dementia patients, outpatient, inpatient, and drug charge related data were used based on the NHI data.

The data used in the current secondary analyses can be accessed on the National Health Insurance Data Sharing Service homepage of NHIS (http://nhiss.nhis.or.kr) according to the data use request [13]. As all data were provided in anonymized and de-identified form, this study qualified for the institutional review board (IRB) review exemption condition [14].

### Prevalence and mortality rate

The prevalence of dementia was defined as the total number of dementia cases based on clinical diagnosis, taken from the NHIS claims data, divided by the total population, obtained from population projections by age and sex of the Korean Statistical Information Service (KOSIS). Korea has a single-payer national health insurance (NHI) system, and claims data include the entire population who use healthcare services. The KOSIS is a national statistical database operated by Statistics Korea. It also produces official statistics to report data for the World Health Organisation (WHO), World Bank, and Organisation for Economic Co-operation and Development (OECD). Dementia cases were based on a primary diagnosis from the NHI claims data according to the national statistics for dementia in South Korea [15] and the Global Dementia Observatory (GDO) of the WHO [16]. To define dementia cases in terms of clinical aspects, we used the International Classification of Disease (ICD)-10 codes Dementia in Alzheimer disease (F00), Vascular dementia (F01), Dementia in other diseases classified elsewhere (F02), Unspecified dementia (F03), and Alzheimer disease (G30) [17–20]. Data on the number of patients with dementia by year, sex, age, number of inpatients and outpatients, number of outpatient visits, hospital length of stay, and drug expenses were used.

The mortality rate of dementia was defined as the number of deaths caused by dementia, based on the cause of death data by sex and age provided by KOSIS, divided by the total population, taken from the KOSIS data of population projections [21].

### Direct costs estimation

Direct costs are goods or services that are actually paid for in the treatment of ‘dementia’ [12]. In this study, direct costs comprised medical care costs, including NHI-covered care costs, care costs not covered by the NHI and co-payments, and the NHI-covered drugs and transportation costs for inpatient and outpatient care. Costs related to the use of long-term care services including nursing home services and home care services were not included in the direct costs. The NHI claims data included patient information according to primary diagnosis, and we calculated the number of patients, length of stay, and costs covered by the NHI. To estimate the medical care costs not covered by the NHI, such as magnetic resonance imaging and ultrasound examinations, we calculated the costs based on the Survey on the Benefit Coverage Rate of National Health Insurance 2019 [22]. It collects hospital cost data annually from sample hospitals using the NHIS. In this survey, 2209 institutions were selected to estimate costs not covered by NHI, which is 2.3% of the total institutions in Korea. Since there are different institutions, such as general hospitals, hospitals, clinics, and pharmacies in Korea, all institutions were stratified into 10 subgroups before sampling to improve sampling accuracy. Then, the Neyman method was used to allocate samples to 25 strata based on the strata variances and similar sampling costs in the strata [23]. The following percentages of inpatient and outpatient care costs not covered by the NHI for dementia care were applied in this analysis: 4.8% in 2015, 5.3% in 2016, 4.7% in 2017, 4.2% in 2018, and 5.8% in 2019, of the total medical expenditure. We regarded transportation costs as costs related to dementia from the Korea Health Panel (KHP), which gathers health service utilisation and healthcare expenditure. In this panel, approximately 8500 households and their family members were surveyed since 2007. The sample for the survey was taken via a 2-stage cluster stratified sampling with probability proportionate to size. In other words, the size of the regional level was first considered, and the households were selected based on the order list at the second stage for sample allocation. The panel data also represents the Korean population [24]. The average transportation cost of all patients was applied to the analysis from the KHP data. All transportation costs were based on round-trip transportation costs; the transportation costs for a patient and a caregiver were included assuming that being accompanied by a guardian when visiting a medical institution was necessary, depending on dementia characteristics.

### Indirect costs estimation

Indirect costs comprise the disease costs, including productivity loss due to medical use and premature death [12]. Opportunity costs were estimated using the human capital approach [19, 25]. The average daily wage of dementia patients for the estimated productivity loss was used for the average wage data by sex and age group from the Wage and Job Information System operated by the Ministry of Employment and Labour. The productivity loss due to medical service use was calculated by multiplying the number of days of hospital stay by the average daily income and dividing by sex and age. For outpatient visits, it was approximately 1/3 of the hospital stay [11, 26]. For premature death, it was calculated assuming that the loss of annual income occurred until the age of 64, based on the age at the time of death. The number of deaths caused by dementia was based on the number of deaths due to ‘Alzheimer’s’ and ‘other dementia’ in the statistics on causes of death by year of Statistics Korea. Caregiver cost is the opportunity cost of the time spent by a caregiver to provide care in the process of using medical services for patients with dementia [12, 27]. Caregiving cost is based on the daily dementia care fee (79 USD/day) published on the Seoul Caregiver Association website. Costs related to social services, including long-term care services and assistive devices used by dementia patients, and informal care costs in daily life other than medical use were not included in the analysis.

### Health-economic costs estimation

The health-economic burden of dementia was estimated by summing the direct and indirect costs and presented separately as the national dementia burden and dementia burden per capita. All calculated costs were converted into US dollars using an exchange rate of one USD = 1138 Korean Won – the average exchange rate during 2015–2019.

## Results

**Fig. 1:**
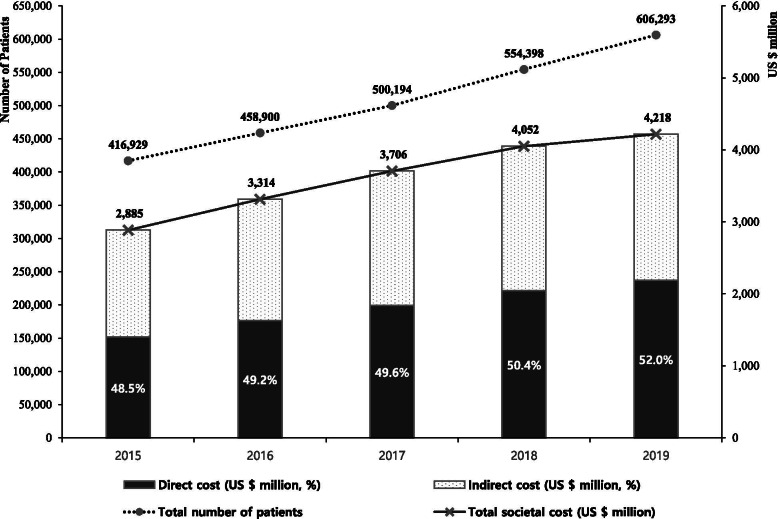
Trends in people with dementia and societal costs for 5 years. NOTE: 1 USD = 1138.32 Korean Won, average exchange rate between 2015 and 2019

The number of patients with dementia in South Korea steadily increased in 2015–2019 by 45.4% (Fig. 1, Table 1), from 416,919 patients in 2015 to 606,293 patients in 2019. The proportion of female patients was approximately 71%, and the sex ratio of patients remained unchanged during the study period. In terms of age, most patients were 75 years or older, approximately increasing by 58.2% from 2015 to 2019 (Table 1).

**Table 1:** Epidemiology of dementia in South Korea from 2015 to 2019

	2015	2016	2017	2018	2019
Total number of patients	416929	458900	500194	554398	606293
Prevalence	0.8	0.9	1.0	1.1	1.2
< 65 years	0.0	0.0	0.0	0.0	0.0
65–74 years	1.9	1.9	1.7	1.7	1.7
≥ 75 years	11.6	12.3	12.8	13.8	14.6
Sex					
Male (%)	119459 (28.7)	131009 (28.5)	142394 (28.5)	158717 (28.6)	174015 (28.7)
Female (%)	297470 (71.3)	327891 (71.5)	357800 (71.5)	395681 (71.4)	432278 (71.3)
Age					
< 65 years (%)	18242 (4.4)	18945 (4.1)	18400 (3.7)	18453 (3.3)	18112 (3.0)
65–74 years (%)	74629 (17.9)	74721 (16.3)	70562 (14.1)	73448 (13.2)	75511 (12.5)
≥ 75 years (%)	324058 (77.7)	365234 (79.6)	411232 (82.2)	462497 (83.4)	512670 (84.6)
Total number of deaths	9458	9160	9290	9738	10352
Mortality (per 100,000)	18.4	17.7	17.9	18.8	20.2
< 65 years	0.3	0.3	0.3	0.4	0.4
65–74 years	14.9	14.2	12.0	10.5	11.8
≥ 75 years	312.8	284.5	269.1	270.9	275.1
Sex					
Male	2823 (29.8)	2850 (31.1)	2699 (29.1)	2980 (30.6)	3113 (30.1)
Female	6635 (70.2)	6310 (68.9)	6591 (70.9)	6758 (69.4)	7239 (69.9)
Age					
< 65 years	127 (1.3)	119 (1.3)	139 (1.5)	176 (1.8)	165 (1.6)
65–74 years	592 (6.3)	572 (6.2)	497 (5.3)	449 (4.6)	534 (5.2)
≥ 65 years	8739 (92.4)	8469 (92.5)	8654 (93.2)	9113 (93.6)	9653 (93.2)

The number of dementia-related deaths has also increased steadily and gradually since 2016. Women accounted for approximately 70% of the deaths, twice that of men. Most deaths were of patients over 75 years, and the mortality rate for patients aged < 65 years was maintained without decreasing, while the mortality rate in other age groups decreased.

The total number of dementia cases increased by 53.8% in 2015–2019 (Table 2). Regarding the types of service, the number of outpatient visits increased continuously and faster than inpatients, while the number of hospitalisations did not change since 2018. The average number of hospital visits per patient was approximately six, which did not change significantly over the same period. The number of visits per female patient was slightly higher than male patients. By age group, the average number of visits per person with dementia aged 65 years and older (6.5 per patient in 2019) was about 1.5 times more than those under 65 (4.9 per patient in 2019).

**Table 2:** Distribution of healthcare utilization by group in South Korea from 2015 to 2019

	2015	2016	2017	2018	2019
Total number of cases (cases)	2490788	2791067	3112271	3490425	3830445
Per capita	6.0	6.1	6.2	6.3	6.3
Type					
Inpatients	662936	752111	840540	911148	920566
Outpatients	1827852	2038956	2271731	2579277	2909879
Sex (per capita)					
Male	661873 (5.5)	736686 (5.6)	814492 (5.7)	917878 (5.8)	1011142 (5.8)
Female	1828915 (6.1)	2054381 (6.3)	2297779 (6.4)	2572547 (6.5)	2819303 (6.5)
Age (per capita)					
< 65 years	81079 (4.4)	85415 (4.5)	85401 (4.6)	88916 (4.8)	88033 (4.9)
65–74 years	393114 (5.3)	399192 (5.3)	380898 (5.4)	396348 (5.4)	411722 (5.5)
≥ 75 years	2016595 (6.2)	2306460 (6.3)	2645972 (6.4)	3005161 (6.5)	3330690 (6.5)
Total days of hospital visits (days)	19612625	22205946	24639589	26593352	27020487
Per capita	47.0	48.4	49.3	48.0	44.6
Type					
Inpatients	17785360	20167712	22368701	24015575	24112718
Outpatients	1827265	2038234	2270888	2577777	2907769
Sex (per capita)					
Male	4414554 (37.0)	4938893 (37.7)	5420597 (38.1)	5831583 (36.7)	5967763 (34.3)
Female	15198071 (51.1)	17267053 (52.7)	19218992 (53.7)	20761769 (52.5)	21052724 (48.7)
Age (per capita)					
< 65 years	452527 (24.8)	468860 (24.7)	486479 (26.4)	498604 (27.0)	472535 (26.1)
65–74 years	1895744 (25.4)	1922681 (25.7)	1833349 (26.0)	1855294 (25.3)	1820096 (24.1)
≥ 75 years	17264354 (53.3)	19814405 (54.3)	22319761 (54.3)	24239454 (52.4)	24727856 (48.2)

During the same period, the total number of hospital visits increased in both males and females, but the average number of hospital visits per patient began to decrease since 2017, resulting in a lower average number of hospital days per capita in 2019 than in 2015 (in men, 37 per patient in 2015 and 34.3 in 2019; in women, 51.1 in 2017 and 48.7 in 2019). Young dementia patients under the age of 65 showed different patterns from other groups in terms of the average number of hospital visits per person. The number of days per capita was relatively short (less than 28 days) and decreased in 2019. However, unlike other groups, the total days of hospital visits per capita in 2019 increased by 5.2% compared to 2015.

The health-economic cost of dementia increased by a factor of 1.5 during the 5 years of the study, with an estimated USD 2885 million in 2015 and USD 4218 million in 2019 (Table 3). Direct costs were 48.5% of total costs in 2015, slightly lower than the share of indirect costs, but continued to increase, accounting for more than 50% of the total costs since 2018. Hospitalisation costs accounted for most direct costs, but outpatient (2.0 times) and drug-related (1.9 times) costs increased the most over 5 years of this study. In particular, the annual growth rate of outpatient and drug-related costs has increased further since 2017; as of 2018 and 2019, they were 26.6 and 20.7% for outpatient costs, and 19.4 and 20.3% for pharmaceutical costs. The productivity loss costs due to hospital visits and premature death were estimated to be USD 28 million and USD 22 million, respectively, in 2019. The total health-economic cost per patient was the highest in 2017 (USD 7410); however, the costs per capita decreased thereafter (USD 6957 in 2019). The health-economic cost per dementia patient aged 75 years and older (USD 7719 in 2015 and USD 7419 in 2019) was the highest among patients; it has decreased gradually since 2017. All results were related to costs of medical use mainly due to dementia, and costs of long-term care and informal care were not included in the analysis.

**Table 3:** Health-economic costs of dementia in South Korea during 2015-2019

	2015	2016	2017	2018	2019
Total health-economic costs (USD million)^a^	2885	3314	3706	4052	4218
Direct costs (USD million, %)	1400 (48.5)	1632 (49.2)	1839 (49.6)	2042 (50.4)	2191 (52.0)
Inpatient	1158 (82.7)	1358 (83.2)	1526 (83.0)	1666 (81.6)	1747 (79.7)
Outpatient	70 (5.0)	79 (4.9)	90 (4.9)	114 (5.6)	137 (6.3)
Drug	138 (9.9)	160 (9.8)	181 (9.8)	216 (10.6)	260 (11.9)
Transportation	33 (2.4)	34 (2.1)	42 (2.3)	46 (2.3)	48 (2.2)
Indirect costs (USD million, %)	1485 (51.5)	1682 (50.8)	1867 (50.4)	2010 (49.6)	2026 (48.0)
Productivity loss due to medical service use	24 (1.6)	25 (1.5)	28 (1.5)	29 (1.4)	28 (1.4)
Productivity loss due to early death	17 (1.2)	19 (1.1)	20 (1.1)	22 (1.1)	22 (1.1)
Family caregivers’ productivity loss	1485 (97.2)	1682 (97.4)	1867 (97.4)	2010 (97.5)	2026 (97.6)
Health-economic costs per capita (USD)	6921	7222	7410	7308	6957
< 65 years	5810	5970	6549	6869	6833
65–74 years	3724	3838	3911	3895	3850
≥ 75 years	7719	7979	8049	7868	7419

^a^1 USD = 1138.32 Korean Won, average exchange rate between 2015 and 2019

## Discussion

The greatest risk factors for dementia are age, genetics, and family history [28–31]. In particular, age is the most prominent factor. Across all OECD countries, around 2% of people aged 65–69 have dementia, compared to more than 40% of those over 90 [32]. However, dementia is not a part of normal ageing. While dementia is a severe loss of cognitive and emotional abilities, which interferes with daily functioning and quality of life, normal ageing may include weakening muscles and bones, stiffening arteries and vessels, and some age-related memory loss [33].

Since the risk of dementia increases with age, it is important to prevent it in advance. Although policy intervention is difficult for age and genetics as risk factors, we can reduce risk by managing other factors. The WHO and OECD recommend physical activity, quitting smoking, and managing chronic diseases including hypertension and diabetes to reduce the risk of dementia [32]. In reality, however, the importance of dementia prevention and management is often overlooked. Indeed, low-income countries are more likely to not have dementia management as a part of the formal care provided by the healthcare system, and dementia management relies on informal care in these countries. Additionally, because dementia care settings vary across countries, it is difficult to accurately measure the full socioeconomic costs of dementia. Therefore, this study estimated the burden of dementia by focusing on health-economic costs, excluding the costs of a family assistance and social services for daily living, to estimate comparable scientific evidence.

In this context, this study estimated the socioeconomic burden of dementia in Korea using nationally representative data. To analyse the burden of dementia, we estimated its prevalence, mortality, and health-economic costs. Between 2015 and 2019, the prevalence of dementia among the elderly aged 65 years or older based on clinical diagnosis increased from 5.9 to 7.3%, with approximately 588000 elderly dementia cases in Korea. Considering that the prevalence of dementia in adults over the age of 65 in Korea was estimated to be about 10.25% (including the elderly who have not been diagnosed with dementia by a doctor and who have been diagnosed with dementia but have not used medical services) [8], the elderly population with dementia who cannot use medical services can be estimated at approximately 2.95%.

As the elderly population has increased, the number of deaths due to dementia has also increased, but the mortality rate for dementia has not changed significantly in the last 5 years. According to OECD health statistics 2021 [34], the age-standardized deaths per 100,000 population for dementia in Korea was 11.3 as of 2017, which was not high compared to the dementia mortality rate in 35 OECD countries. However, according to the cause of death statistics of KOSIS, the number of deaths from dementia is increasing every year along with the rapid increase in the elderly population. In particular, considering that only deaths due to Alzheimer’s disease, vascular dementia and unspecified dementia are counted in the death statistic of KOSIS, the actual number of deaths for dementia in Korea may be higher than the statistics.

The total number of dementia treatment cases increased by 53.8% between 2015 and 2019. By type of service, the number of outpatient visits continuously increased compared to inpatient services, and the number of hospitalisations has not changed since 2018. The average number of hospitalisations per capita was also about six, which did not change significantly over the same period.

The total health-economic cost of dementia has increased by about 1.5 times in the last 5 years and was estimated to be about USD 4218 million. Direct costs were 52.0% in 2019, and its proportion has been steadily increasing over the past 5 years; hospitalisation and drug costs were the majority of direct costs. In particular, inpatient costs increased by about 50.8% during the same period, whereas outpatient costs increased by 95.0% and drug costs by 88.0%. The main reasons for the increase in direct costs of dementia patients are costs of outpatient and drug utilisation. In addition, increased direct costs for people with dementia was similar to the increased overall medical expenditure for the elderly in South Korea. According to the annual report of NHIS, medical expenditure for elderly aged 65 or older increased by about 64% between 2015 and 2019.

Alternatively, indirect costs accounted for 47.9% of the total burden, mainly from family members and caregivers. Specifically, the productivity loss cost due to medical use increased by 16%, and productivity loss due to death was 26.9%. However, due to the disease mechanism of dementia, the cost of productivity loss of patients accounts for a low share of indirect costs, most of it attributed to the opportunity cost of family caregivers. Nevertheless, caregiving costs increased at a relatively low rate of 36.9% compared to medical costs because the labour costs of caregivers in Korea are still very low. The health-economic costs per capita for dementia have continuously increased since 2015 and decreased in 2019, estimated at approximately USD 6957. Specifically, health-economic costs per capita due to dementia increased by 17.6% for those under the age of 65, but there was little change for those over 65. Since the majority of dementia patients are 65 years or older, it seems that the health-economic costs per capita have not changed significantly over the past 5 years.

Korea’s health-economic burden of dementia per capita (excluding costs for daily assistance and nursing home) is similar to previous studies conducted in countries with similar dementia management settings [17, 33, 35–37]. When converted to USD based on purchasing power parity for proper cross-country comparison, the socioeconomic burden per capita due to dementia in Korea was approximately USD 9200. The burden of dementia per capita in Korea was somewhat lower than in Germany (USD 13168) and France (USD 10381), which have high access to medical services with the social health insurance system. However, it was higher than the health-economic burden caused by dementia in Switzerland (USD 6392), which provides a private health insurance system with a very high co-payment.

As this study focused on the annual health-economic costs of dementia, the results were much lower than a previous study [10]. According to Han et al. [10], the total social costs of dementia per person, including the long-term care services for dementia patients, were about USD 23877. As of 2016, about 39% of 457524 dementia patients were long-term care insurance beneficiaries, and one-third of the total direct cost per person (USD 4071) was related to long-term care service use. In their study, direct medical costs per person were USD 9466, and there was a significant difference from the results of this study (USD 3556 per person direct cost in 2016). The difference in the direct costs related to medical service use is attributed to the difference in the criteria for dementia patients. While the current study included only cases where dementia is the main purpose of medical use, Han et al. [10] analysed all medical service costs by dementia patients.

Ageing is a global mega-trend, and an increasing population with dementia is predicted, causing a great socioeconomic burden for most countries. Increased elderly population leads to a surge in medical costs. Dementia is a typical degenerative disease where not only medical expenditure but also social expenses for care occur at the same time. Korea has a significant socioeconomic burden due to dementia [10]. Dementia involves a large proportion of indirect costs, such as caregiving, in addition to direct costs. In this study, the caregiving costs proportion was approximately 47% of the total health-economic costs. Given Korea’s demographic trends and dementia burden, health policies for dementia prevention and management should be prioritised.

Dementia management not only includes treatment and social care for dementia patients but also dementia prevention for healthy people and diagnosis of risk groups [38]. The Korean government announced the national responsibility for dementia in 2017 as a countermeasure against the surge in related social burden. According to the plan, the scope of support for dementia prevention, medical diagnosis, and appropriate treatment, as well as social care, that is, long-term care service support for dementia patients was expanded. Through this study, which analysed the health-economic cost trends due to dementia over the past 5 years, it was possible to indirectly confirm changes in dementia medical expenses before and after the government’s dementia policy expansion. The study is significant because we investigated aspects that were not analysed in the previous study [10] that comprehensively analysed the total social cost of dementia.

This study can be a good example for accurately estimating the health-economic burden of dementia using reliable and representative data. Nevertheless, there were some limitations. First, the health-economic burden of dementia may have been underestimated because the study utilised the primary diagnosis based on the ICD-10 codes by doctors to estimate the dementia prevalence and mortality rates. We excluded cases where the doctor diagnosed dementia as a secondary diagnosis. The national statistics for dementia in South Korea [15] also announced the statistics of healthcare service utilization of patients with dementia based on the primary diagnosis according to the GDO of WHO [16]. Nevertheless, the result of current study should be interpreted as the partial estimate of dementia care costs because we only included the cases where dementia was the primary diagnosis and excluded the other care costs related to the dementia including long-term care services and assistive devices. Second, dementia has a significant socioeconomic cost as the disease severity increases [39]. However, in this study, the burden of disease severity could not be estimated. We used the ICD-10 code of the health insurance claims data to analyse more accurate national statistical data. Future research could be conducted to determine the socioeconomic burden of dementia by disease severity using the Clinical Dementia Rating and Mini-Mental State Examination.

## Conclusion

This study estimated the health-economic burden of dementia in South Korea. The burden of dementia in Korea is already considerable and is expected to increase alongside the elderly population in the future. Dementia has a high proportion of indirect costs, such as caregiving costs, as well as direct costs including medical treatment. Health policies addressing the prevention and management of dementia should be prioritised.

## Data Availability

Requests for more detailed information regarding the study can be addressed to the corresponding author. The secondary administrative data used in the current study were available from the National Statistical Office and the Wage and Job Information System operated by the Ministry of Employment and Labour. The epidemiological data obtained from the NHI claims data and KHP data were available from the KHP website.

## Abbreviations

GDO: Global Dementia Observatory
ICD: International Classification of Disease
KHP: The Korea Health Panel
KOSIS: The Korean Statistical Information Service
LTCI: Long-term Care Insurance
NHI: National Health Insurance
NHIS: National Health Insurance Service
OECD: Organisation for Economic Co-operation and Development
USD: US dollar
WHO: World Health Organisation

## References

[1] Geldmacher DS, Whitehouse PJ (1996). Evaluation of dementia. N Engl J Med.

[2] World Health Organization. Dementia. https://www.who.int/news-room/fact-sheets/detail/dementia. Accessed 29 May 2021.

[3] Gustavsson A, Brinck P, Bergvall N, Kolasa K, Wimo A, Winblad B (2011). Predictors of costs of care in Alzheimer’s disease: a multinational sample of 1222 patients. Alzheimers Dement.

[4] Wimo A, Guerchet M, Ali GC, Wu YT, Prina AM, Winblad B (2017). The worldwide costs of dementia 2015 and comparisons with 2010. Alzheimers Dement.

[5] Alzheimer Disease International (2010). World Alzheimer Report 2010: the global economic impact of dementia.

[6] OECD (2018). Working better with age: Korea, ageing and employment policies.

[7] Statistics Korea (2019). Population status and prospects of the world and Korea - reflecting the special projection for the future population in 2019.

[8] Ministry of Health and Welfare (2020). The 4th national dementia plan.

[9] Kim H, Kwon S (2021). A decade of public long-term care insurance in South Korea: policy lessons for aging countries. Health Policy.

[10] Han E-J, Lee J, Cho E, Kim H (2021). Socioeconomic costs of dementia based on utilization of health care and long-term-care services: a retrospective cohort study. Int J Environ Res Public Health.

[11] Shon C, Choi H-Y, Shim J-J, Park S-Y, Lee KS, Yoon S-J (2016). The economic burden of hepatitis A, B, and C in South Korea. Jpn J Infect Dis.

[12] Davidson M, Beeri MS (2000). Cost of Alzheimer’s disease. Dialogues Clin Neurosci.

[13] Seong S, Kim Y-Y, Park S, Khang Y, Kim H, Park J (2017). Cohort profile: the National Health Insurance Service-National Health Screening Cohort (NHIS_HEALS) in Korea. BMJ Open.

[14] Korea National Institute for Bioethics Policy. http://www.irb.or.kr/UserMenu01/Exemption.aspx. Accessed 17 June 2021.

[15] Ministry of Health and Welfare, National Institue of Dementia (2020). Korean dementia observatory 2019.

[16] World Health Organization. The global dementia observatory reference guide. Geneva: World Health Organization; 2018. https://www.who.int/publications/i/item/who-msd-mer-18.1. Accessed 9 Aug 2021.

[17] Jönsson L, Jönhagen ME, Kilander L, Soininen H, Hallikainen M, Waldemar G (2006). Determinants of costs of care for patients with Alzheimer’s disease. Int J Geriatr Psychiatry.

[18] Kang IO, Lee SY, Kim SY, Park CY (2007). Economic cost of dementia patients according to the limitation of the activities of daily living in Korea. Int J Geriatr Psychiatry.

[19] Kraft E, Marti M, Werner S, Sommer H (2010). Cost of dementia in Switzerland. Swiss Med Wkly.

[20] Schwarzkopf L, Menn P, Leidl R, Wunder S, Mehlig H, Marx P (2012). Excess costs of dementia disorders and the role of age and gender - an analysis of German health and long-term care insurance claims data. BMC Health Serv Res.

[21] Causes of Death Statistics (2020). Korean statistical information service.

[22] National Health Insurance Service (2021). 2019 survey on the benefit coverage rate of National Health Insurance.

[23] Bankier MD (1988). Power allocations: determining sample sizes for subnational areas. Am Stat.

[24] Korea Health Panel Study (2020). Survey.

[25] Drummond MF, Sculpher MJ, Claxton K, Stoddart GL, Torrance GW. Methods for the economic evaluation of health care programmes. Oxford: Oxford University Press; 2015.

[26] Oh I-H, Yoon S-J, Seo H-Y, Kim E-J, Kim YA (2011). The economic burden of musculoskeletal disease in Korea: a cross sectional study. BMC Musculoskelet Disord.

[27] Chang H-S, Kim H-J, Nam C-M, Lim S-J, Jang Y-H, Kim S (2012). The socioeconomic burden of coronary heart disease in Korea. J Prev Med Public Health.

[28] Fratiglioni L, Ahlbom A, Viitanen M, Winblad B (1993). Risk factors for late-onset Alzheimer’s disease: a population-based, case-control study. Ann Neurol.

[29] Hebert L, Bienias J, Aggarwal N, Wilson R, Bennett D, Shah R (2010). Change in risk of Alzheimer disease over time. Neurology.

[30] Mayeux R, Sano M, Chen J, Tatemichi T, Stern Y (1991). Risk of dementia in first-degree relatives of patients with Alzheimer’s disease and related disorders. Arch Neurol.

[31] Saunders AM, Strittmatter WJ, Schmechel D, George-Hyslop PS, Pericak-Vance MA, Joo S (1993). Association of apolipoprotein E allele ϵ4 with late-onset familial and sporadic Alzheimer’s disease. Neurology.

[32] Organization for Economic Co-operation Development (2017). Dementia prevalence. Health at a glance 2017.

[33] Centers for Disease Control and Prevention (2019). What is dementia?.

[34] Organization for Economic Co-operation Development (2021). Health status: causes of mortality, OECD health statistics 2021.

[35] Rapp T, Andrieu S, Molinier L, Grand A, Cantet C, Mullins CD (2012). Exploring the relationship between Alzheimer’s disease severity and longitudinal costs. Value Health.

[36] Schaller S, Mauskopf J, Kriza C, Wahlster P, Kolominsky-Rabas PL (2015). The main cost drivers in dementia: a systematic review. Int J Geriatr Psychiatry.

[37] Schwarzkopf L, Menn P, Kunz S, Holle R, Lauterberg J, Marx P (2011). Costs of care for dementia patients in community setting: an analysis for mild and moderate disease stage. Value Health.

[38] Moise P, Schwarzinger M, Um M-Y, the Dementia Experts’ Group (2004). Dementia care in 9 OECD countries: a comparative analysis.

[39] Mesterton J, Wimo A, Langworth S, Winblad B, Jonsson L (2010). Cross sectional observational study on the societal costs of Alzheimer’s disease. Curr Alzheimer Res.

